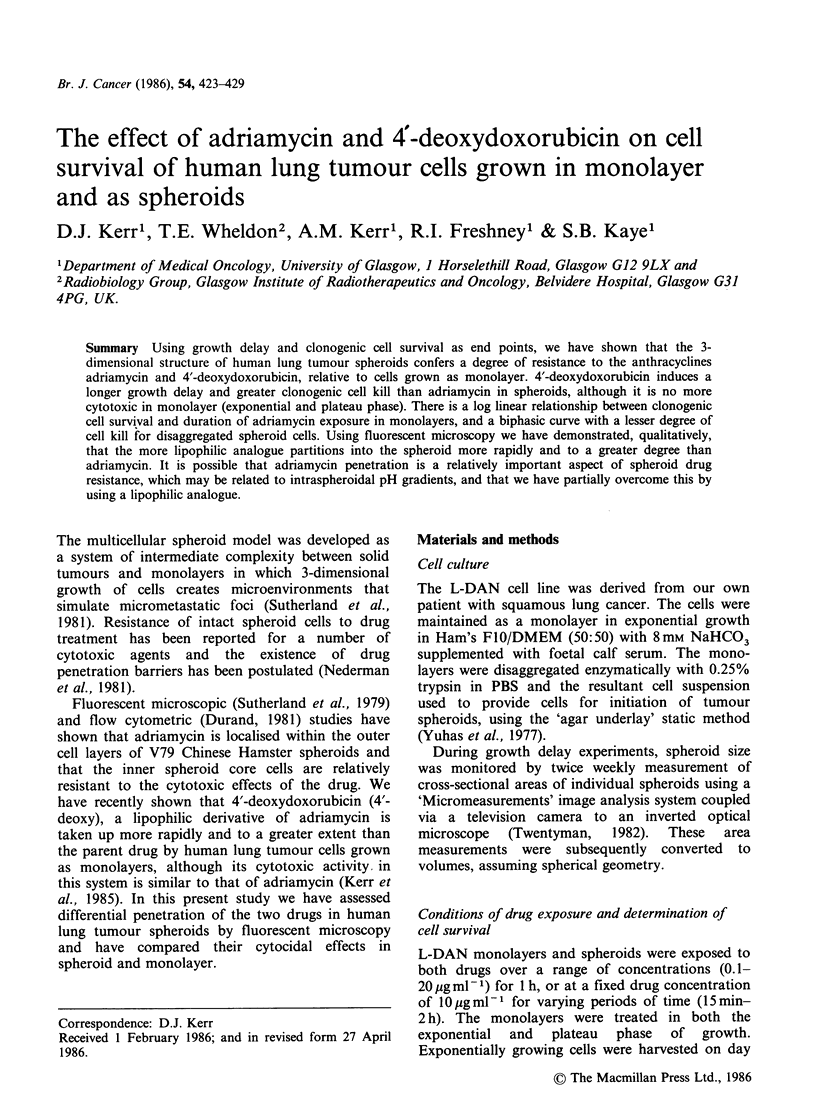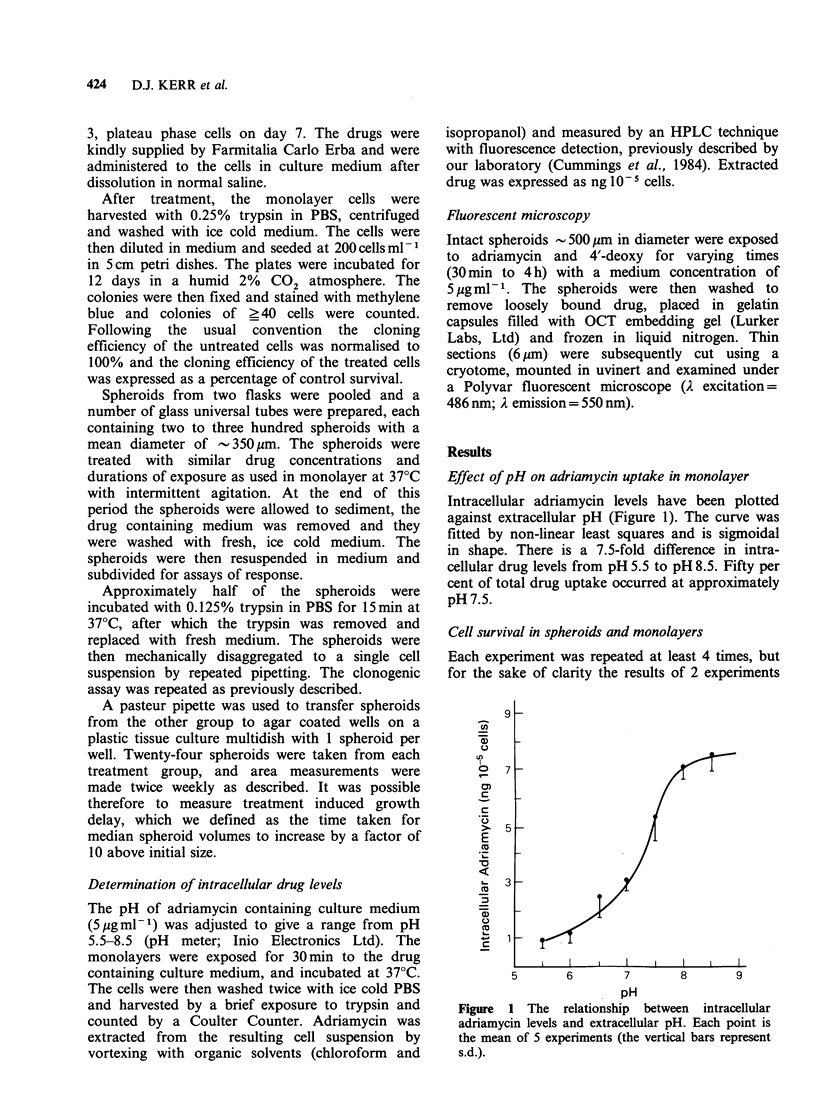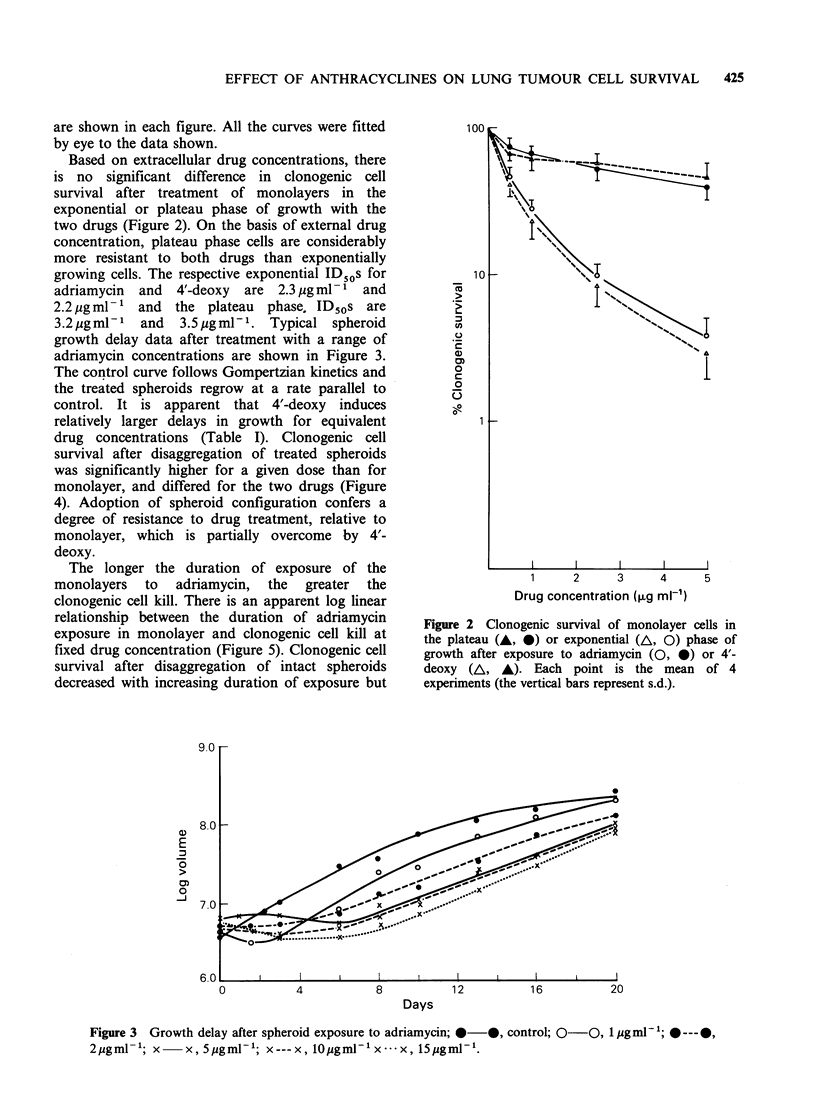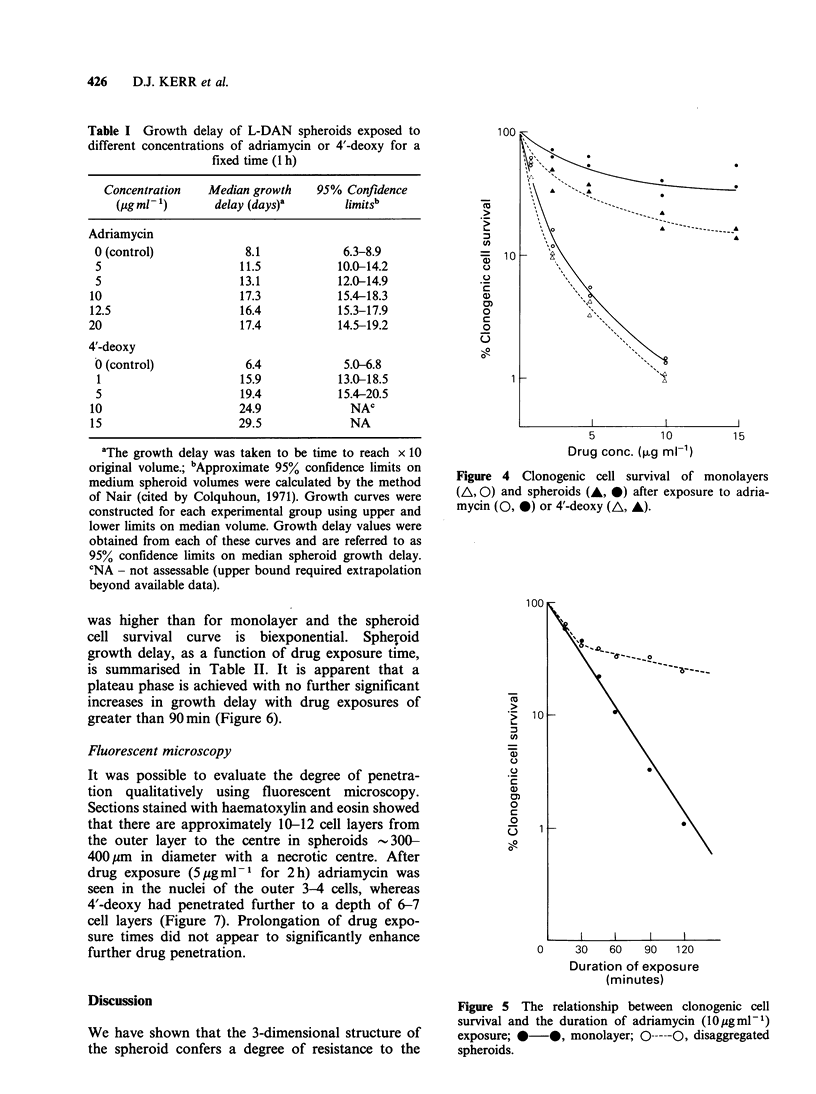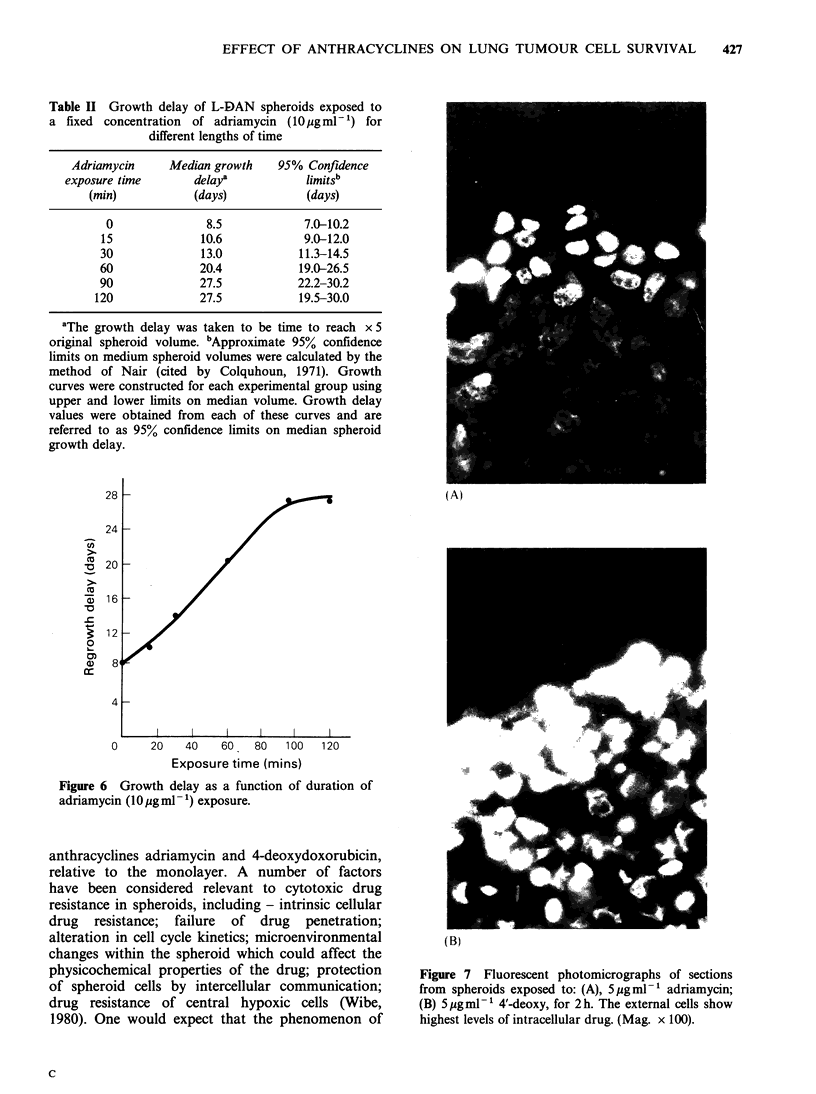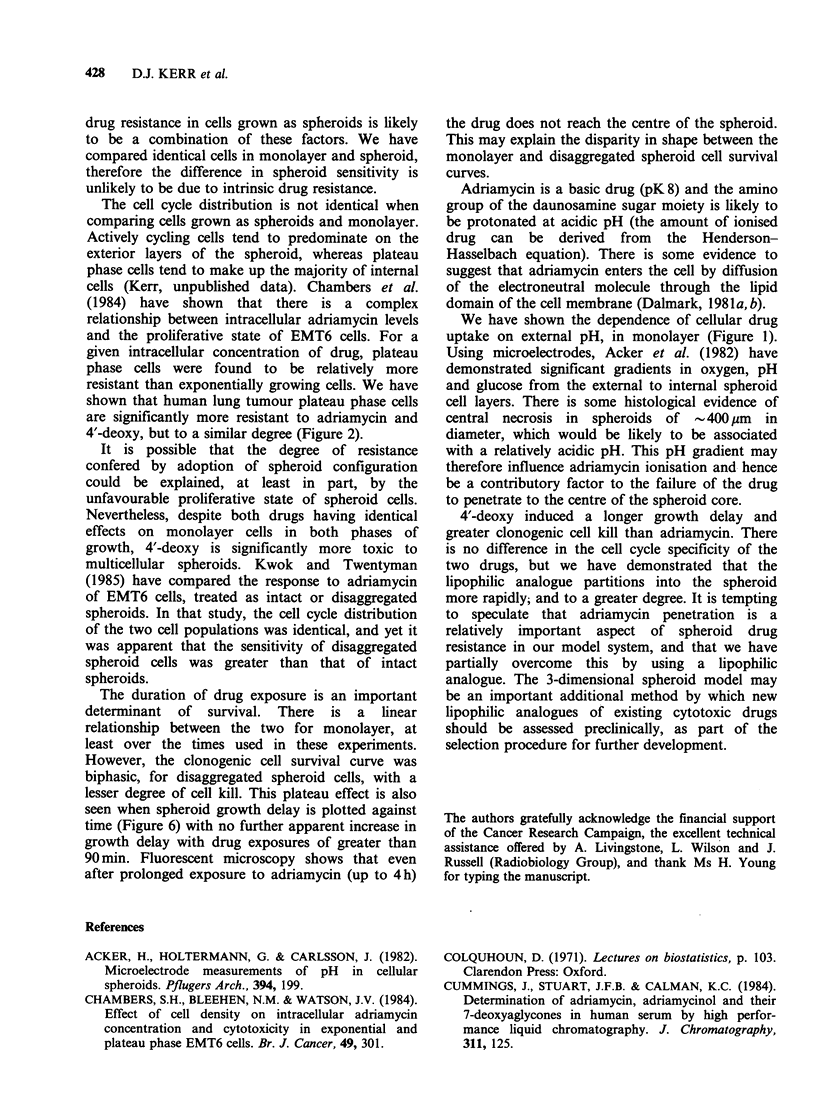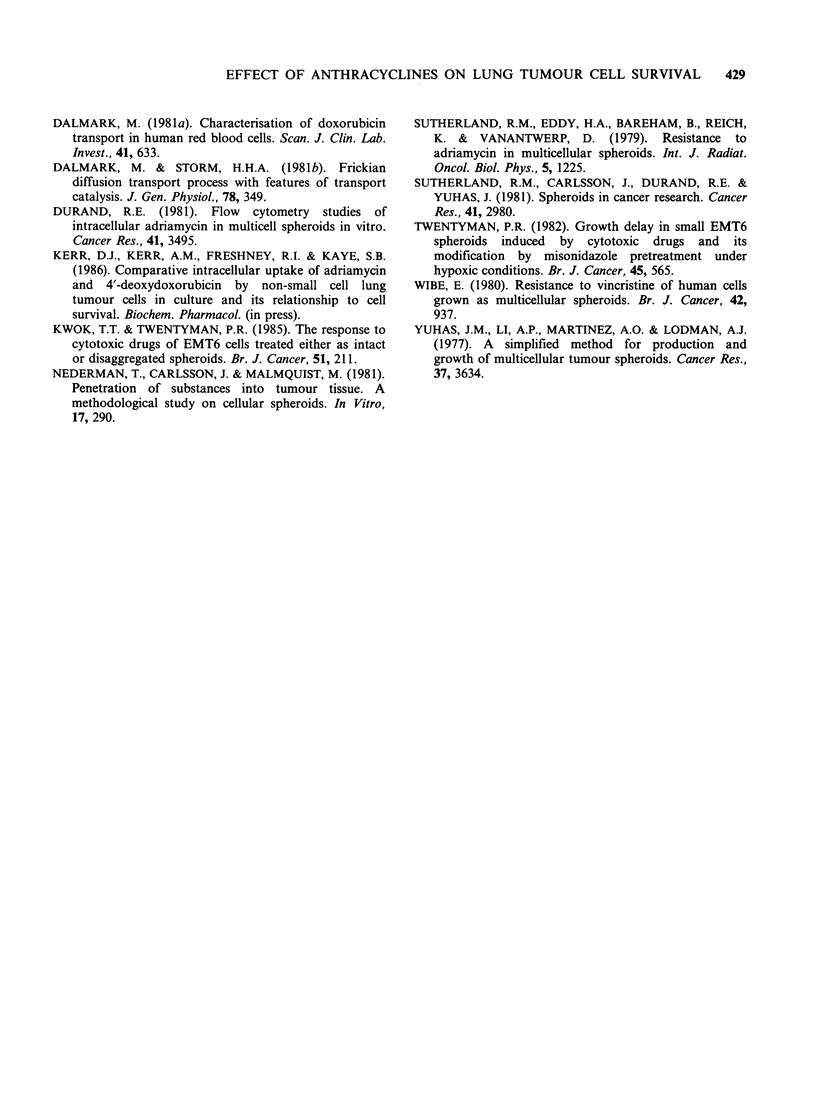# The effect of adriamycin and 4'-deoxydoxorubicin on cell survival of human lung tumour cells grown in monolayer and as spheroids.

**DOI:** 10.1038/bjc.1986.193

**Published:** 1986-09

**Authors:** D. J. Kerr, T. E. Wheldon, A. M. Kerr, R. I. Freshney, S. B. Kaye

## Abstract

**Images:**


Br. J. Cancer (1986), **54**, 423–429

### The effect of adriamycin and 4′-deoxydoxorubicin on cell survival of human lung tumour cells grown in monolayer and as spheroids

D.J. Kerr[1], T.E. Wheldon[2], A.M. Kerr[1], R.I. Freshney[1] & S.B. Kaye[1]

[1]*Department of Medical Oncology, University of Glasgow, 1 Horselethill Road, Glasgow G12 9LX and*
[2]*Radiobiology Group, Glasgow Institute of Radiotherapeutics and Oncology, Belvidere Hospital, Glasgow G31 4PG, UK.*

**Summary** Using growth delay and clonogenic cell survival as end points, we have shown that the 3-dimensional structure of human lung tumour spheroids confers a degree of resistance to the anthracyclines adriamycin and 4′-deoxydoxorubicin, relative to cells grown as monolayer. 4′-deoxydoxorubicin induces a longer growth delay and greater clonogenic cell kill than adriamycin in spheroids, although it is no more cytotoxic in monolayer (exponential and plateau phase). There is a log linear relationship between clonogenic cell survival and duration of adriamycin exposure in monolayers, and a biphasic curve with a lesser degree of cell kill for disaggregated spheroid cells. Using fluorescent microscopy we have demonstrated, qualitatively, that the more lipophilic analogue partitions into the spheroid more rapidly and to a greater degree than adriamycin. It is possible that adriamycin penetration is a relatively important aspect of spheroid drug resistance, which may be related to intraspheroidal pH gradients, and that we have partially overcome this by using a lipophilic analogue.

The multicellular spheroid model was developed as a system of intermediate complexity between solid tumours and monolayers in which 3-dimensional growth of cells creates microenvironments that simulate micrometastatic foci (Sutherland *et al.*, 1981). Resistance of intact spheroid cells to drug treatment has been reported for a number of cytotoxic agents and the existence of drug penetration barriers has been postulated (Nederman *et al.*, 1981).

Fluorescent microscopic (Sutherland *et al.*, 1979) and flow cytometric (Durand, 1981) studies have shown that adriamycin is localised within the outer cell layers of V79 Chinese Hamster spheroids and that the inner spheroid core cells are relatively resistant to the cytotoxic effects of the drug. We have recently shown that 4′-deoxydoxorubicin (4′-deoxy), a lipophilic derivative of adriamycin is taken up more rapidly and to a greater extent than the parent drug by human lung tumour cells grown as monolayers, although its cytotoxic activity in this system is similar to that of adriamycin (Kerr *et al.*, 1985). In this present study we have assessed differential penetration of the two drugs in human lung tumour spheroids by fluorescent microscopy and have compared their cytocidal effects in spheroid and monolayer.

Correspondence: D.J. Kerr
Received 1 February 1986; and in revised form 27 April 1986.

### Materials and methods

### *Cell culture*

The L-DAN cell line was derived from our own patient with squamous lung cancer. The cells were maintained as a monolayer in exponential growth in Ham's F10/DMEM (50:50) with 8 mM $NaHCO_3$ supplemented with foetal calf serum. The monolayers were disaggregated enzymatically with 0.25% trypsin in PBS and the resultant cell suspension used to provide cells for initiation of tumour spheroids, using the 'agar underlay' static method (Yuhas *et al.*, 1977).

During growth delay experiments, spheroid size was monitored by twice weekly measurement of cross-sectional areas of individual spheroids using a 'Micromeasurements' image analysis system coupled via a television camera to an inverted optical microscope (Twentyman, 1982). These area measurements were subsequently converted to volumes, assuming spherical geometry.

### *Conditions of drug exposure and determination of cell survival*

L-DAN monolayers and spheroids were exposed to both drugs over a range of concentrations (0.1–20 $\mu g\,ml^{-1}$) for 1 h, or at a fixed drug concentration of 10 $\mu g\,ml^{-1}$ for varying periods of time (15 min–2 h). The monolayers were treated in both the exponential and plateau phase of growth. Exponentially growing cells were harvested on day

© The Macmillan Press Ltd., 1986

3, plateau phase cells on day 7. The drugs were kindly supplied by Farmitalia Carlo Erba and were administered to the cells in culture medium after dissolution in normal saline.

After treatment, the monolayer cells were harvested with 0.25% trypsin in PBS, centrifuged and washed with ice cold medium. The cells were then diluted in medium and seeded at 200 cells $ml^{-1}$ in 5 cm petri dishes. The plates were incubated for 12 days in a humid 2% $CO_2$ atmosphere. The colonies were then fixed and stained with methylene blue and colonies of $\geqq 40$ cells were counted. Following the usual convention the cloning efficiency of the untreated cells was normalised to 100% and the cloning efficiency of the treated cells was expressed as a percentage of control survival.

Spheroids from two flasks were pooled and a number of glass universal tubes were prepared, each containing two to three hundred spheroids with a mean diameter of $\sim 350\,\mu m$. The spheroids were treated with similar drug concentrations and durations of exposure as used in monolayer at 37°C with intermittent agitation. At the end of this period the spheroids were allowed to sediment, the drug containing medium was removed and they were washed with fresh, ice cold medium. The spheroids were then resuspended in medium and subdivided for assays of response.

Approximately half of the spheroids were incubated with 0.125% trypsin in PBS for 15 min at 37°C, after which the trypsin was removed and replaced with fresh medium. The spheroids were then mechanically disaggregated to a single cell suspension by repeated pipetting. The clonogenic assay was repeated as previously described.

A pasteur pipette was used to transfer spheroids from the other group to agar coated wells on a plastic tissue culture multidish with 1 spheroid per well. Twenty-four spheroids were taken from each treatment group, and area measurements were made twice weekly as described. It was possible therefore to measure treatment induced growth delay, which we defined as the time taken for median spheroid volumes to increase by a factor of 10 above initial size.

*Determination of intracellular drug levels*

The pH of adriamycin containing culture medium ($5\,\mu g\,ml^{-1}$) was adjusted to give a range from pH 5.5–8.5 (pH meter; Inio Electronics Ltd). The monolayers were exposed for 30 min to the drug containing culture medium, and incubated at 37°C. The cells were then washed twice with ice cold PBS and harvested by a brief exposure to trypsin and counted by a Coulter Counter. Adriamycin was extracted from the resulting cell suspension by vortexing with organic solvents (chloroform and isopropanol) and measured by an HPLC technique with fluorescence detection, previously described by our laboratory (Cummings *et al.*, 1984). Extracted drug was expressed as ng $10^{-5}$ cells.

*Fluorescent microscopy*

Intact spheroids $\sim 500\,\mu m$ in diameter were exposed to adriamycin and 4′-deoxy for varying times (30 min to 4 h) with a medium concentration of $5\,\mu g\,ml^{-1}$. The spheroids were then washed to remove loosely bound drug, placed in gelatin capsules filled with OCT embedding gel (Lurker Labs, Ltd) and frozen in liquid nitrogen. Thin sections ($6\,\mu m$) were subsequently cut using a cryotome, mounted in uvinert and examined under a Polyvar fluorescent microscope ($\lambda$ excitation = 486 nm; $\lambda$ emission = 550 nm).

### Results

*Effect of pH on adriamycin uptake in monolayer*

Intracellular adriamycin levels have been plotted against extracellular pH (Figure 1). The curve was fitted by non-linear least squares and is sigmoidal in shape. There is a 7.5-fold difference in intracellular drug levels from pH 5.5 to pH 8.5. Fifty per cent of total drug uptake occurred at approximately pH 7.5.

*Cell survival in spheroids and monolayers*

Each experiment was repeated at least 4 times, but for the sake of clarity the results of 2 experiments

**Figure 1** The relationship between intracellular adriamycin levels and extracellular pH. Each point is the mean of 5 experiments (the vertical bars represent s.d.).

are shown in each figure. All the curves were fitted by eye to the data shown.

Based on extracellular drug concentrations, there is no significant difference in clonogenic cell survival after treatment of monolayers in the exponential or plateau phase of growth with the two drugs (Figure 2). On the basis of external drug concentration, plateau phase cells are considerably more resistant to both drugs than exponentially growing cells. The respective exponential $ID_{50}$s for adriamycin and 4′-deoxy are 2.3 $\mu g\,ml^{-1}$ and 2.2 $\mu g\,ml^{-1}$ and the plateau phase, $ID_{50}$s are 3.2 $\mu g\,ml^{-1}$ and 3.5 $\mu g\,ml^{-1}$. Typical spheroid growth delay data after treatment with a range of adriamycin concentrations are shown in Figure 3. The control curve follows Gompertzian kinetics and the treated spheroids regrow at a rate parallel to control. It is apparent that 4′-deoxy induces relatively larger delays in growth for equivalent drug concentrations (Table I). Clonogenic cell survival after disaggregation of treated spheroids was significantly higher for a given dose than for monolayer, and differed for the two drugs (Figure 4). Adoption of spheroid configuration confers a degree of resistance to drug treatment, relative to monolayer, which is partially overcome by 4′-deoxy.

The longer the duration of exposure of the monolayers to adriamycin, the greater the clonogenic cell kill. There is an apparent log linear relationship between the duration of adriamycin exposure in monolayer and clonogenic cell kill at fixed drug concentration (Figure 5). Clonogenic cell survival after disaggregation of intact spheroids decreased with increasing duration of exposure but

**Figure 2** Clonogenic survival of monolayer cells in the plateau (▲, ●) or exponential (△, ○) phase of growth after exposure to adriamycin (○, ●) or 4′-deoxy (△, ▲). Each point is the mean of 4 experiments (the vertical bars represent s.d.).

**Figure 3** Growth delay after spheroid exposure to adriamycin; ●—●, control; ○—○, 1 $\mu g\,ml^{-1}$; ●---●, 2 $\mu g\,ml^{-1}$; ×—×, 5 $\mu g\,ml^{-1}$; ×---×, 10 $\mu g\,ml^{-1}$ ×···×, 15 $\mu g\,ml^{-1}$.

**Table I** Growth delay of L-DAN spheroids exposed to different concentrations of adriamycin or 4′-deoxy for a fixed time (1 h)

| *Concentration* ($\mu g\, ml^{-1}$) | *Median growth delay (days)*[a] | *95% Confidence limits*[b] |
|---|---|---|
| Adriamycin | | |
| 0 (control) | 8.1 | 6.3–8.9 |
| 5 | 11.5 | 10.0–14.2 |
| 5 | 13.1 | 12.0–14.9 |
| 10 | 17.3 | 15.4–18.3 |
| 12.5 | 16.4 | 15.3–17.9 |
| 20 | 17.4 | 14.5–19.2 |
| 4′-deoxy | | |
| 0 (control) | 6.4 | 5.0–6.8 |
| 1 | 15.9 | 13.0–18.5 |
| 5 | 19.4 | 15.4–20.5 |
| 10 | 24.9 | NA[c] |
| 15 | 29.5 | NA |

[a]The growth delay was taken to be time to reach ×10 original volume.; [b]Approximate 95% confidence limits on medium spheroid volumes were calculated by the method of Nair (cited by Colquhoun, 1971). Growth curves were constructed for each experimental group using upper and lower limits on median volume. Growth delay values were obtained from each of these curves and are referred to as 95% confidence limits on median spheroid growth delay. [c]NA – not assessable (upper bound required extrapolation beyond available data).

was higher than for monolayer and the spheroid cell survival curve is biexponential. Spheroid growth delay, as a function of drug exposure time, is summarised in Table II. It is apparent that a plateau phase is achieved with no further significant increases in growth delay with drug exposures of greater than 90 min (Figure 6).

*Fluorescent microscopy*

It was possible to evaluate the degree of penetration qualitatively using fluorescent microscopy. Sections stained with haematoxylin and eosin showed that there are approximately 10–12 cell layers from the outer layer to the centre in spheroids ~300–400 $\mu$m in diameter with a necrotic centre. After drug exposure (5 $\mu g\, ml^{-1}$ for 2 h) adriamycin was seen in the nuclei of the outer 3–4 cells, whereas 4′-deoxy had penetrated further to a depth of 6–7 cell layers (Figure 7). Prolongation of drug exposure times did not appear to significantly enhance further drug penetration.

### Discussion

We have shown that the 3-dimensional structure of the spheroid confers a degree of resistance to the

**Figure 4** Clonogenic cell survival of monolayers (△, ○) and spheroids (▲, ●) after exposure to adriamycin (○, ●) or 4′-deoxy (△, ▲).

**Figure 5** The relationship between clonogenic cell survival and the duration of adriamycin (10 $\mu g\, ml^{-1}$) exposure; ●——●, monolayer; ○----○, disaggregated spheroids.

**Table II** Growth delay of L-DAN spheroids exposed to a fixed concentration of adriamycin ($10 \mu g ml^{-1}$) for different lengths of time

| *Adriamycin exposure time (min)* | *Median growth delay*[a] *(days)* | *95% Confidence limits*[b] *(days)* |
|---|---|---|
| 0 | 8.5 | 7.0–10.2 |
| 15 | 10.6 | 9.0–12.0 |
| 30 | 13.0 | 11.3–14.5 |
| 60 | 20.4 | 19.0–26.5 |
| 90 | 27.5 | 22.2–30.2 |
| 120 | 27.5 | 19.5–30.0 |

[a]The growth delay was taken to be time to reach ×5 original spheroid volume. [b]Approximate 95% confidence limits on medium spheroid volumes were calculated by the method of Nair (cited by Colquhoun, 1971). Growth curves were constructed for each experimental group using upper and lower limits on median volume. Growth delay values were obtained from each of these curves and are referred to as 95% confidence limits on median spheroid growth delay.

**Figure 6** Growth delay as a function of duration of adriamycin ($10 \mu g ml^{-1}$) exposure.

anthracyclines adriamycin and 4-deoxydoxorubicin, relative to the monolayer. A number of factors have been considered relevant to cytotoxic drug resistance in spheroids, including – intrinsic cellular drug resistance; failure of drug penetration; alteration in cell cycle kinetics; microenvironmental changes within the spheroid which could affect the physicochemical properties of the drug; protection of spheroid cells by intercellular communication; drug resistance of central hypoxic cells (Wibe, 1980). One would expect that the phenomenon of

**Figure 7** Fluorescent photomicrographs of sections from spheroids exposed to: (A), $5 \mu g ml^{-1}$ adriamycin; (B) $5 \mu g ml^{-1}$ 4′-deoxy, for 2 h. The external cells show highest levels of intracellular drug. (Mag. ×100).

drug resistance in cells grown as spheroids is likely to be a combination of these factors. We have compared identical cells in monolayer and spheroid, therefore the difference in spheroid sensitivity is unlikely to be due to intrinsic drug resistance.

The cell cycle distribution is not identical when comparing cells grown as spheroids and monolayer. Actively cycling cells tend to predominate on the exterior layers of the spheroid, whereas plateau phase cells tend to make up the majority of internal cells (Kerr, unpublished data). Chambers *et al.* (1984) have shown that there is a complex relationship between intracellular adriamycin levels and the proliferative state of EMT6 cells. For a given intracellular concentration of drug, plateau phase cells were found to be relatively more resistant than exponentially growing cells. We have shown that human lung tumour plateau phase cells are significantly more resistant to adriamycin and 4′-deoxy, but to a similar degree (Figure 2).

It is possible that the degree of resistance confered by adoption of spheroid configuration could be explained, at least in part, by the unfavourable proliferative state of spheroid cells. Nevertheless, despite both drugs having identical effects on monolayer cells in both phases of growth, 4′-deoxy is significantly more toxic to multicellular spheroids. Kwok and Twentyman (1985) have compared the response to adriamycin of EMT6 cells, treated as intact or disaggregated spheroids. In that study, the cell cycle distribution of the two cell populations was identical, and yet it was apparent that the sensitivity of disaggregated spheroid cells was greater than that of intact spheroids.

The duration of drug exposure is an important determinant of survival. There is a linear relationship between the two for monolayer, at least over the times used in these experiments. However, the clonogenic cell survival curve was biphasic, for disaggregated spheroid cells, with a lesser degree of cell kill. This plateau effect is also seen when spheroid growth delay is plotted against time (Figure 6) with no further apparent increase in growth delay with drug exposures of greater than 90 min. Fluorescent microscopy shows that even after prolonged exposure to adriamycin (up to 4 h) the drug does not reach the centre of the spheroid. This may explain the disparity in shape between the monolayer and disaggregated spheroid cell survival curves.

Adriamycin is a basic drug (pK 8) and the amino group of the daunosamine sugar moiety is likely to be protonated at acidic pH (the amount of ionised drug can be derived from the Henderson–Hasselbach equation). There is some evidence to suggest that adriamycin enters the cell by diffusion of the electroneutral molecule through the lipid domain of the cell membrane (Dalmark, 1981*a, b*).

We have shown the dependence of cellular drug uptake on external pH, in monolayer (Figure 1). Using microelectrodes, Acker *et al.* (1982) have demonstrated significant gradients in oxygen, pH and glucose from the external to internal spheroid cell layers. There is some histological evidence of central necrosis in spheroids of ~400 μm in diameter, which would be likely to be associated with a relatively acidic pH. This pH gradient may therefore influence adriamycin ionisation and hence be a contributory factor to the failure of the drug to penetrate to the centre of the spheroid core.

4′-deoxy induced a longer growth delay and greater clonogenic cell kill than adriamycin. There is no difference in the cell cycle specificity of the two drugs, but we have demonstrated that the lipophilic analogue partitions into the spheroid more rapidly; and to a greater degree. It is tempting to speculate that adriamycin penetration is a relatively important aspect of spheroid drug resistance in our model system, and that we have partially overcome this by using a lipophilic analogue. The 3-dimensional spheroid model may be an important additional method by which new lipophilic analogues of existing cytotoxic drugs should be assessed preclinically, as part of the selection procedure for further development.

The authors gratefully acknowledge the financial support of the Cancer Research Campaign, the excellent technical assistance offered by A. Livingstone, L. Wilson and J. Russell (Radiobiology Group), and thank Ms H. Young for typing the manuscript.

### References

ACKER, H., HOLTERMANN, G. & CARLSSON, J. (1982). Microelectrode measurements of pH in cellular spheroids. *Pflugers Arch.*, **394**, 199.

CHAMBERS, S.H., BLEEHEN, N.M. & WATSON, J.V. (1984). Effect of cell density on intracellular adriamycin concentration and cytotoxicity in exponential and plateau phase EMT6 cells. *Br. J. Cancer*, **49**, 301.

COLQUHOUN, D. (1971). *Lectures on biostatistics*, p. 103. Clarendon Press: Oxford.

CUMMINGS, J., STUART, J.F.B. & CALMAN, K.C. (1984). Determination of adriamycin, adriamycinol and their 7-deoxyaglycones in human serum by high performance liquid chromatography. *J. Chromatography*, **311**, 125.

DALMARK, M. (1981*a*). Characterisation of doxorubicin transport in human red blood cells. *Scan. J. Clin. Lab. Invest.*, **41**, 633.

DALMARK, M. & STORM, H.H.A. (1981*b*). Frickian diffusion transport process with features of transport catalysis. *J. Gen. Physiol.*, **78**, 349.

DURAND, R.E. (1981). Flow cytometry studies of intracellular adriamycin in multicell spheroids in vitro. *Cancer Res.*, **41**, 3495.

KERR, D.J., KERR, A.M., FRESHNEY, R.I. & KAYE, S.B. (1986). Comparative intracellular uptake of adriamycin and 4′-deoxydoxorubicin by non-small cell lung tumour cells in culture and its relationship to cell survival. *Biochem. Pharmacol.* (in press).

KWOK, T.T. & TWENTYMAN, P.R. (1985). The response to cytotoxic drugs of EMT6 cells treated either as intact or disaggregated spheroids. *Br. J. Cancer*, **51**, 211.

NEDERMAN, T., CARLSSON, J. & MALMQUIST, M. (1981). Penetration of substances into tumour tissue. A methodological study on cellular spheroids. *In Vitro*, **17**, 290.

SUTHERLAND, R.M., EDDY, H.A., BAREHAM, B., REICH, K. & VANANTWERP, D. (1979). Resistance to adriamycin in multicellular spheroids. *Int. J. Radiat. Oncol. Biol. Phys.*, **5**, 1225.

SUTHERLAND, R.M., CARLSSON, J., DURAND, R.E. & YUHAS, J. (1981). Spheroids in cancer research. *Cancer Res.*, **41**, 2980.

TWENTYMAN, P.R. (1982). Growth delay in small EMT6 spheroids induced by cytotoxic drugs and its modification by misonidazole pretreatment under hypoxic conditions. *Br. J. Cancer*, **45**, 565.

WIBE, E. (1980). Resistance to vincristine of human cells grown as multicellular spheroids. *Br. J. Cancer*, **42**, 937.

YUHAS, J.M., LI, A.P., MARTINEZ, A.O. & LODMAN, A.J. (1977). A simplified method for production and growth of multicellular tumour spheroids. *Cancer Res.*, **37**, 3634.